# The House-Governor Makes a Defence

**Published:** 1918-09-28

**Authors:** E. W. Morris

**Affiliations:** House-Governor. London Hospital, Whitechapel, E. 1


### Some London Hospital Correspondents.

[Correspondence on all subjects is invited, but we cannot in any way be responsible for the opinions expressed by our correspondents, who must give their name and address as a guarantee of good faith, but not necessarily for publication. Correspondents are reminded that brevity of style and conciseness of statement greatly facilitate early insertion.]

### The House-Governor Makes a Defence.

*To the Editor of* THE HOSPITAL.

SIR,—I will give Major Chapple every credit for his desire to benefit the nursing profession in his attempts to improve the salary, the hours, and the condition of service of nurses. There are many of us who have been intimately connected with nursing and nurses for many years who are quite as eager that the lot of the nurse shall be a better one, but we feel keen resentment that unworthy means should be adopted to achieve that ambition. I venture to say that Major Chapple's letter is an instance of such unworthy means. It states truths, but it does not state all the truth, and it speaks in innuendo and suggestion.

He makes the statement that probationers sign on for four years—two years' training and two years' service. He might have mentioned that if they pay for their training, as many do, at the rate of £1 1s. a week, we providing board and lodging, they do not sign any agreement. The agreement applies to those who do not pay for training. He also omitted to say that these are given a small salary by the hospital, even during the two years of their training, £17 and £24.

It is also true, as he says, that at the end of the two years they get a certificate of two years' training. Then he makes the assertion, "This is of no service to the nurse; it cannot be used by her, and only serves to enable the London Hospital to advertise in the *Times*: "Trained nurses can be had immediately."

It is not quite clear what he means by this. It may not be of use to her while she still remains at the hospital—she does not need it. But to say that it is of no use to her when she leaves the hospital is simply untrue. Hundreds and hundreds of London Hospital nurses know it to be untrue; many are holding influential and honourable appointments which they obtained by the help of this certificate. And when he says that we are giving this certificate simply that we can advertise in the *Times*, he is also saying what is untrue, and what he knows is untrue.

The London Hospital issued certificates for two years' training long before it had a staff of nurses which were sent out to nurse private cases, and for years when other hospitals were issuing certificates for one year's training.

Major Chapple believes that no nurse can be trained under three years. He is quite entitled to that opinion. The House Committee of the London Hospital is equally entitled to its opinion that the "London" can train in two years, partly because of its size and wealth of material, but much more because of the organisation of the training—the preliminary seven weeks' coaching at a separate institution, where she is taught how to make a bed with a patient in it, names of instruments, etc., before a nurse enters the ward at all; the lectures, the classes, etc. When and if the House Committee consider a longer term than two years is advisable, I am quite sure they will decide to give it without the slightest hesitation.

The next statement by Major Chapple, stating that we pay nurses in their third year 13s. 5d. a week, while they earn £2 12s. 6d. a week for us, whereby we make a profit of £5,000 a year, is falsely true and very truly false. It is false because it is only part of the truth. Yes, we pay a "private staff" nurse in her third year after entering the hospital £35 a year, and it is true that while she is nursing a case in private she earns for the hospital two and a half guineas a week. But she is not nursing such a case all the time, and her pay goes on whether she is nursing or not. And £35 does not represent all her pay. She receives her board, lodging, washing, and part uniform as well; she is paid during her holidays, paid during sickness (full salary for a year, or more if necessary), and after eighteen years of service receives full pay for life. This is paid whatever new work she takes up, in whatever part of the world. Your readers will see, therefore, that to state that we pay private nurses 13s. 5d. a week and receive £2 12s. 6d. was an unfair statement. It was true, but not nearly all the truth. He omitted so much of the truth that his statement almost becomes a lie.

I am in entire agreement with him that nurses are shamefully underpaid. I would like to see every certificated nurse earn at least £100 a year. But this end cannot be attained all at once, and meanwhile nothing is gained by inaccurate statements. To suggest that an honourable group of men, such as the House Committee of the London Hospital, "sweat" and "exploit" nurses because a profit of £5,000 a year is made, is an unworthy thing to say. These gentlemen give more than that to the hospital out of their own pockets, often anonymously.

The sending out of nurses to private cases is part of a definite policy. It provides skilled nursing to those who from their financial position are ineligible for admission to hospital. I know that there are some on the committee who would prefer no profit whatever were made, and Lord Knutsford, who is in the North of Scotland and knows nothing of this correspondence, has told me that he personally wished that we could do this work at actual cost and without profit. The profit amounts to over £15 and under £20 for each of the nurses engaged, after deducting expenses. But whatever profit is made is more than eaten up by the cost of training and boarding of the nurses in the hospital, and if there were any surplus it would go to the nursing and treatment of the sick poor in our wards. Why should I blush when I admit that the profit on nursing the sick rich is spent on nursing the sick poor?

I am with Major Chapple entirely when he asks for better salary, shorter hours, more off-duty time; and I believe that all these things are coming. They will take time. Shorter hours mean larger staff; larger staff means more accommodation; more accommodation means building and equipment. This is to be faced, and is being faced. But nothing is gained by writing about "defenceless and sweated women"; the nurses themselves do not like it, for they still regard their work as a mission, and not simply a trade.—Yours faithfully,

E. W. MORRIS, House-Governor.

London Hospital, Whitechapel, E. 1,
September 24, 1918.

